# Cross-cultural adaptation and psychometric validation of the STarT back tool for Jordanian Arabic-speaking adults with low back pain

**DOI:** 10.1371/journal.pone.0336398

**Published:** 2025-11-06

**Authors:** Owis Eilayyan, Thamer A. Altaim, Alaa Salameh, Fadi M. Al Zoubi

**Affiliations:** 1 Department of Physical Therapy, Faculty of Allied Medical Sciences, Al-Ahliyya Amman University, Amman, Jordan; 2 Department of Physical Therapy, Faculty of Applied Medical Sciences, The Hashemite University, Zarqa, Jordan; 3 Department of Rehabilitation Sciences, The Hong Kong Polytechnic University, Kowloon, Hong Kong SAR, China; Uttara Adhunik Medical College, BANGLADESH

## Abstract

**Background:**

The Keele STarT Back Tool (STarTBack) was developed to categorize people with low back pain based on disability risk. The tool was cross-culturally adapted and validated in different languages and countries, including Arabic in Saudi Arabia. However, the tool has not been cross-culturally adapted and validated among Arabic-Jordanian speakers.

**Objective:**

To cross-culturally adapt and validate the Keele STarT Back Tool (STarTBack) for Arabic-speaking adults with low back pain (LBP) in Jordan.

**Methods:**

This prospective cross-sectional study was conducted in hospitals and physical therapy departments. The STarTBack was translated following international guidelines. Cross-cultural adaptation was assessed through interviews with experts and individuals with LBP. Internal consistency, construct validity (via correlation with related measures), and discriminative validity (using Receiver Operating Characteristic curves) were examined.

**Results:**

Twenty participants participated in the content validity assessment (mean age: 41.3 years; 50% female), while 107 participants took part in the pre-final version testing (mean age: 39.2 years; 54.2% female). One item required minor modification for clarity. Our preliminary results showed that the adapted STarTBack-AR demonstrated good internal consistency (Cronbach’s α = 0.73). Moderate-to-high correlations supported construct validity. Discriminative validity was acceptable-to-excellent for disability, catastrophizing, anxiety and depression.

**Conclusion:**

The culturally adapted STarTBack-AR is a reliable and valid tool for stratifying Arabic-speaking Jordanian patients with LBP according to their risk of disability. Its implementation has potential to improve care through targeted treatment approaches, thereby reducing the risk of disability.

## Introduction

Low back pain (LBP) is one of the most prevalent conditions globally, affecting approximately 568.4 million individuals worldwide in 2019 [1]. Since 1990, it has been the leading cause of years lived with disability (YLDs), posing a significant public health concern [2]. In Jordan, there were approximately 717,146 reported cases of LBP, resulting in 81,779 YLDs [3]. Most patients seeking primary care for LBP suffer from non-specific LBP, which lacks an identifiable pathological origin [4]. This condition not only causes physical discomfort but also leads to psychosocial and economic burdens, limiting physical and social functions, causing depression and anxiety, and negatively impacting overall health and quality of life [5,6].

Clinical practice guidelines for managing LBP recommend a variety of interventions, including multidisciplinary approaches such as medical and physiotherapy treatment, psychotherapy, and medications [7]. Although numerous studies have evaluated the effectiveness of these treatments [8,9], results often show minimal differences in outcomes [8,9]. This disparity may arise from the diverse limitations experienced by individuals with LBP, suggesting that a one-size-fits-all treatment approach is inadequate. Instead, tailored treatments for individuals with similar characteristics may yield better results [10,11].

Stratified care is an effective approach that categorizes patients based on shared characteristics and limitations, facilitating more personalized treatment plans [12]. This method enhances clinical decision-making and improves the quality of care, ultimately maximizing patient health outcomes [12]. Clinicians recognize the existence of various patient subgroups with non-specific LBP, highlighting the need for stratified care [13]. Research supports that this approach not only improves patient health outcomes but is also a cost-effective treatment strategy [14–16].

Several stratified care approaches are used in clinical practice, including prognosis-based stratification, treatment responsiveness, and classification based on underlying mechanisms [17]. One such approach, developed by Keele University, categorizes individuals with LBP into three risk groups: low, medium, and high. The Keele STarT Back Tool (STarTBack) was designed to classify patients with LBP based on their risk of disability [18]; it is a validated tool and commonly used tool for stratifying patients with LBP. This self-reported questionnaire consists of nine items aimed at predicting physical and psychological disability in individuals with LBP [18].

The STarTBack tool has been proven to be reliable and valid and has been cross-culturally adapted and validated in various languages [19] and countries, including an Arabic version in Saudi Arabia [20]. However, it has not yet been adapted or validated for Arabic-speaking Jordanians, and the Saudi version cannot be generalized to all LBP patients, as it was validated only for those receiving tertiary care. Thus, cross-cultural adaptation of patient-reported outcomes is essential for maintaining the accuracy and reliability of a measure across diverse languages and cultural contexts [21].

Translating and adapting the STarTBack tool for Jordanian Arabic speakers could improve clinical practice in Jordan by helping clinicians identify subgroups of patients with LBP and deliver appropriate treatment for each subgroup. By implementing individualized treatment, clinicians can improve clinical outcomes, reduce the burden of LBP, and improve the health and quality of life of individuals with LBP in Jordan. Therefore, the aims of this study are to: 1) linguistically and culturally adapt the STarTBack tool; and 2) assess the psychometric properties (reliability and validity) of the translated version for use in adults with LBP in Jordan.

## Materials and methods

### Study design

This research employed a prospective, cross-sectional design divided into two phases: the first focused on the cross-cultural adaptation of the STarTBack tool, while the second phase assessed the psychometric properties of the translated version. Ethical approval was obtained from the Research Ethics Board of Al-Ahliyya Amman University (AAU/1/2/2023-2024). In phase 1 (October 24 to November 5, 2023), verbal informed consent was audio-recorded and obtained from all participants. In phase 2 (November 5, 2023 to July 24, 2024), electronic informed consent was obtained from all participants, where participants read the consent form online and agreed to participate in the study.

### Phase 1: Cross-cultural adaptation

The STarTBack tool consists of nine items divided into two subscales: physical and psychosocial. Most items are rated as either “agree” or “disagree”, with scores ranging from 0 to 9, where a higher score indicates a worse prognosis. The psychosocial subscale includes items 5–9, where a score of 5 indicates a high risk for poor outcome [18].

Permission was obtained from the original developer to adapt the STarTBack tool for Arabic speakers in Jordan. The adaptation followed the guidelines set by Beaton et al. (2000) [22], which include six stages:

*Stage 1: Initial Translation*: Two independent translators created independently separate Arabic versions of the STarTBack—one (T1) with medical knowledge and another (T2) without.

*Stage 2: Synthesis of the Translations*: The two translators, along with an observer (primary investigator), synthesized the translations to produce a common version (T12).

*Stage 3: Back Translation*: Two independent translators, fluent in English but without a medical background, translated T12 back into English, creating two back-translated versions (BT1 and BT2).

*Stage 4: Expert Committee*: An expert committee reviewed all translations and reached a consensus, ensuring the final version was comprehensible to individuals with a reading level of around 12 years. The committee included three methodologists, two health professionals, one language professional, and the translators. The results of the expert committee meeting and the translations were shared with the developer.

*Stage 5: Test of the Pre-final Version*: The pre-final version was tested for clarity among 20 Arabic-speaking participants with LBP. The participants at this stage were asked to complete the STarTBack and then attended recorded interviews with probing questions about the meaning of each item in the STarTBack and the difficulties or ambiguities of the item’s wording. The psychometric properties are discussed in detail in the second phase of this study.

*Stage 6: Submission of Documentation to the Developers*: All documentation and adapted translations were submitted to the original developer for appraisal.

### Phase 2: Psychometric assessment

*Participants*: were adults aged 18–60 with non-specific LBP, recruited from Jordan. Inclusion criteria included a history of non-specific LBP lasting one month or more and proficiency in Arabic, while pregnant individuals were excluded.

*Procedure*: The research team orally contacted heads of physiotherapy clinics across Jordan to identify individuals who met the eligibility criteria. Eligible participants who expressed interest in the study received an online package containing sociodemographic information and self-administered questionnaires, which took approximately 15 minutes to complete. The package (S1 File) included the Arabic version of the STarTBack tool, the Oswestry Disability Index (ODI) [23], the Pain Catastrophizing Scale (PCS) [24], the Fear-Avoidance Beliefs Questionnaire (FABQ) [25], the Numerical Pain Rating Scale (NPRS) [26], and the Hospital Anxiety and Depression Scale (HADS) [27]. Permissions were obtained to use the aforementioned questionnaires.

*Reliability*: Internal reliability of the Arabic version of the STarTBack tool (STarTBack-AR) was assessed using Cronbach’s alpha (α), with a value above 0.7 indicates acceptable reliability [28,29].

*Construct Validity*: This was evaluated through convergent and discriminative validity.

Convergent validity was assessed by correlating the overall score and the psychosocial subscale of the STarTBack-AR with similar constructs, including disability (ODI), pain (NPRS), anxiety and depression (HADS), catastrophizing (PCS), and physical activity of the FABQ. Pearson correlation coefficients were calculated between the total score of the STarTBack-AR and the scores of NPRS, ODI, HADS, FABQ, and PCS. The strength of the correlations was classified as follows: high (*r* > 0.7), moderate (0.5 < *r* < 0.7), low (0.3 < *r* < 0.5), and negligible (0 < *r* < 0.3) [30]. We expected significant moderate-to-high correlations between the total score of STarTBack-AR, its psychosocial subscale, and the other questionnaires.

Discriminative validity was assessed using the areas under the curve (AUC) for various measures: ODI, HADS, FABQ, and PCS. The AUC was employed to determine the discriminative validity of the STarTBack-AR. The cut-off points for each measure were as follows: ODI > 20 [23], FABQ-PA > 8 [31], HADS ≥ 8 [32], and PCS ≥ 30 [33]. The discriminative value was classified as follows: 0.7 < AUC < 0.8 is considered “acceptable discrimination,” 0.8 < AUC < 0.9 is considered “excellent discrimination,” and AUC ≥ 0.9 is considered “outstanding discrimination” [34].

*Data Analysis and Sample Size*: Participant characteristics were analyzed descriptively using SAS software. A sample size of 100 participants was deemed sufficient for psychometric assessments. The literature recommends a minimum of 100 participants to estimate the Cronbach’s alpha [35]. The sample size required to estimate the construct validity was determined using the equations outlined by Douglas G. Bonett and Thomas A. Wright (2000) [36]. These equations depend on the power, type I error (α), expected Pearson correlation, and Fisher coefficient. Assuming a power of 0.8, type I error (α) of 0.05, and with an expected correlation range from 0.2–0.8, a sample of 100 participants is adequate for correlation estimation. Age and pain duration were the only variables with missing values (2%). Therefore, missing values were not replaced [37].

*Floor/Ceiling Effects*: These effects were evaluated to determine the tool’s responsiveness, defined as more than 15% of participants scoring at the extremes (either lowest or highest) [38].

## Results

**Fig 1:**
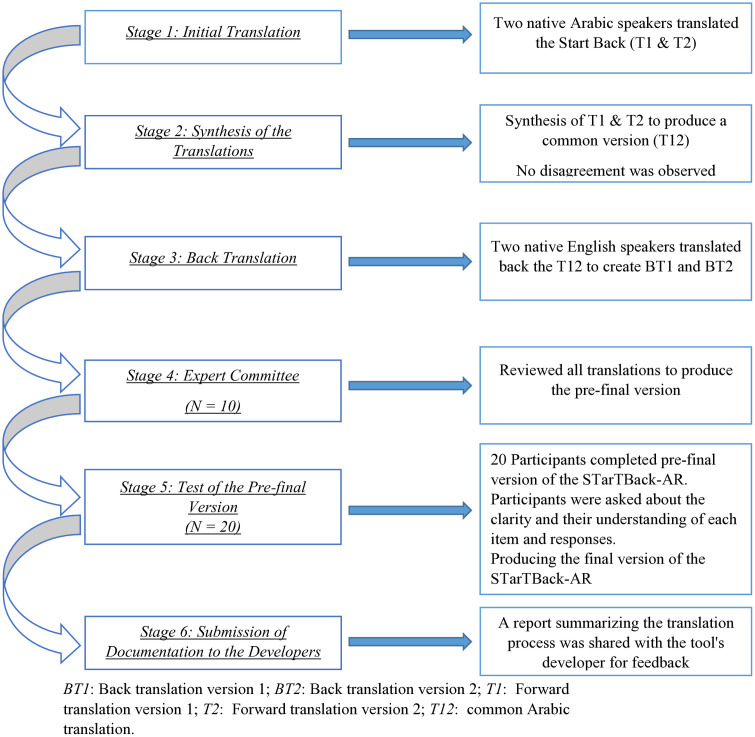
The stages of cross-cultural adaptation of the Arabic version of Start Back tool. It outlines the detailed steps we followed to adapt the Start Back tool into Arabic.

The results are presented according to the stages of validation of the STarTBack-AR, and are summarized in Fig 1 [39].

### Stages 1–4: Cross cultural adaptation

Two fluent Arabic speakers independently translated the original STarTBack into Arabic. The first translator (AS) held a Master’s degree in physiotherapy, while the second translator held a Bachelor’s degree in English. Both were also fluent in English. The translators and primary investigator (OE) reviewed the two Arabic versions and found no significant differences. A common Arabic version was then developed and sent to two native English speakers for back-translation into English. An expert committee, comprising the authors and clinical experts, reviewed all translations and reached consensus on item wording, with no significant differences between versions noted. A report summarizing the translation process was shared with the tool’s developer for feedback. S2 File includes the commonly translated version.

### Stage 5 A: Test of the pre-final version (Content validity)

Twenty Arabic-speaking patients with LBP (mean age, 41.3 years, SD = 9.2; 50% female) completed the pre-final STarTBack–AR and attended individual interviews to assess the content validity. All items achieved a clarity score above 9 out of 10, except Item 5 (*It’s not really safe for a person with a condition like mine to be physically active*), scored 8.67/10. The clarity scores were 10/10 for two items: 7 (*I feel that my back pain is terrible and it’s never going to get any better*) and 9 (*Overall, how bothersome has your back pain been in the last 2 weeks?*). The clarity of the items’ response options was 10/10 for all items.

We made changes to one item only (item 5), while no modifications were made to the other items. Participants suggested changes that were not related to the items’ wording itself (Table 1). Item 5 was unclear to the first five participants, who stated a problem in the phrasing. Therefore, we changed the items from “it is not safe” to “it is dangerous”. Then, the modified item was presented again to three participants who reviewed the original item and re-asked them about its clarity, they declared that the revised version became clearer than the first form. Furthermore, we showed the two forms of the item to all upcoming participants, and they mentioned that the modified version was clearer than the first version.

**Table 1:** Content validity of STarTBack–Arabic.

Item	Item’s clarity	Item responses clarity	Difficult words	Suggested changes
1	9.6	10	No (20/20)	Make the sentence as a question Do not specify the time frame
2	9.95	10	No (20/20)	No
3	9.47	10	No (20/20)	Specify the place where I walk Use long distance instead of short distance Determine what the short distance is Make it as question and specify the time frame The distance I walked is less than usual
4	9.86	10	No (20/20)	Dressing with difficulty or more time I couldn’t wear my cloths as usual Add “As usual”
5	8.67	10	No (18/20)	
6	9.65	10	No (20/20)	Giving an example of warrying thoughts Make it as a question Add “because of LBP”
7	10	10	No (20/20)	Make it as a question
8	9.95	10	No (20/20)	Add “because of LBP” Add “LBP prevents me performing what I used to do”
9	10	10	No (20/20)	No

### Stage 5 B: Test of the pre-final version (Reliability and construct validity)

One hundred and seven patients (mean age 39.2 years, SD = 11.4; 54.2% female) were recruited. The average pain intensity was 2.8 (SD = 2.1), and the average duration of symptoms lasting for 44.6 months (ranged 1–240 months). The mean total and psychosocial subscale of the STarTBack–AR scores were 4.16 (SD = 2.4) and 2.12 (SD = 1.6), respectively. Regarding management of LBP, 54 participants (50.5%) had received physical therapy, 34 (31.8%) had used pain medications, and 28 (26.2%) had consulted a specialist physician. Table 2 presents the participants’ characteristics. The majority of participants sought combined physical and pharmacological interventions for LBP management. Overall, participants experienced chronic or recurrent LBP of long duration, reflecting the target patient population for the STarTBack tool.

**Table 2:** Baseline characteristics of the participants.

Variable	M (sd), N (%)
Age	39.2 (11.4)
Gender, female	58 (54.2%)
Pain duration (months)	44.7 (50.2)
STARTBACK – overall score	4.16 (2.4)
STARTBACK – psychosocial subscale	2.12 (1.6)
STARTBACK – physical subscale	2 (1.2)
Pain intensity (NPS)	2.8 (2.1)
Physical disability (ODI)	23.5 (13.1)
Pain catastrophizing (PCS)	22.5 (12.9)
Depression (HADS)	6.74 (3.3)
Anxiety (HADS)	8.1 (4)
Employment status	
Employed	79 (73.8%)
Homemaker	20 (18.7%)
Unemployed	3 (2.8%)
Retired	2 (1.9%)
Student	3 (2.8%)

HADS: Hospital Anxiety and Depression Scale; NPS: Numerical Pain Scale; ODI: Oswestry Disability Index; PCS: Pain Catastrophizing Scale.

#### Internal reliability.

Cronbach’s alpha was calculated to assess internal reliability. The overall scale demonstrated good reliability (**α = 0.73**) as did the psychosocial subscale (**α = 0.71**). (Table 3).

**Table 3:** Cronbach’s alpha values of the overall and psychosocial scores of STarTBack–Arabic.

	Cronbach’s alpha (95%CI)
Overall Score	0.73 (0.65-0.8)
Psychosocial Subscale	0.71 (0.62-0.79)

CI: Confidence Interval.

#### Construct validity (Convergent).

Pearson’s correlations examined convergent validity between the STarTBack–AR scores and established measures of pain, physical function, and psychosocial factors. The total score of STarTBack–AR correlated strongly with the ODI score **(*r* = 0.75)**; moderately with the PCS score **(*r* = 0.61)**; and low with anxiety **(r = 0.49)**, depression **(*r* = 0.37)**, and pain score **(*r* = 0.4)**.

The psychosocial subscale also showed moderate correlation with the ODI (*r* = 0.7) and PCS score (r = 0.61), and low with anxiety (*r* = 0.49), depression (*r* = 0.37), and pain score (*r* = 0.38) (Table 4).

**Table 4:** Pearson correlations between the overall and psychosocial scores of STarTBack–Arabic with other health outcomes.

	Overall Score	Psychosocial Subscale
ODI	0.74 (0.61-0.87)	0.7 (0.55-0.83)
FABQ-PA	0.3 (0.12-0.49)	0.38 (0.2-0.56)
Catastrophizing (PCS)	0.61 (0.45-0.76)	0.6 (0.44-0.75)
Depression (HADS)	0.41 (0.23-0.58)	0.4 (0.22-0.58)
Anxiety (HADS)	0.5 (0.34-0.67)	0.47 (0.3-0.64)
Pain Intensity (NPS)	0.4 (0.23-0.58)	0.4 (0.22-0.58)

HADS: Hospital Anxiety and Depression Scale; NPS: Numerical Pain Scale; ODI: Oswestry Disability Index; PCS: Pain Catastrophizing Scale.

#### Construct validity (Discriminative).

Table 5 presents the AUC values used to examine the discriminative validity of the total and psychosocial subscale scores of STarTBack–AR against established measures of ODI, FABQ-PA, HADS (depression and anxiety), and PCS.

**Table 5:** Area under the receiver operating characteristic curve (AUC) for the overall scores and psychosocial subscale scores against reference standard at baseline.

Reference	Case definition	Overall Score AUC (95% CI)	Psychosocial Subscale AUC (95% CI)
ODI	> 20	0.88 (0.8-0.95)	0.84 (0.76-0.92)
FABQ-PA	> 8	0.71 (0.62-0.81)	0.76 (0.66-0.86)
Catastrophizing (PCS)	≥ 30	0.82 (0.73-0.9)	0.84 (0.77-0.92)
Depression (HADS)	> 7	0.72 (0.62-0.82)	0.72 (0.62-0.82)
Anxiety (HADS)	> 7	0.75 (0.65-0.84)	0.74 (0.65-0.84)

AUC: Area Under Curve; CI: Confidence Interval; HADS: Hospital Anxiety and Depression Scale; NPS: Numerical Pain Scale; ODI: Oswestry Disability Index; PCS: Pain Catastrophizing Scale.

The total score AUC values ranged from **0.71** (95%CI, 0.62–0.81) to **0.88** (95%CI, 0.8–0.95), indicating acceptable to excellent discriminative ability. The psychosocial subscale AUCs ranged from 0.72 (95% CI, 0.62–0.82) to 0.84 (95% CI, 0.76–0.92), demonstrating acceptable to excellent discrimination.

Specifically, the STarTBack–AR total score and psychosocial subscale scores demonstrated excellent discriminative ability by the physical disability and pain catastrophizing.

### Floor/ceiling effects

There was no signiﬁcant ﬂoor or ceiling effects of STarTBack–AR; because only two participants (1.9%) scored lowest and four participants (3.7%) scored highest.

## Discussion

This study aimed to cross-cultural adapt and validate the STarT back tool for Arabic-speaking adults with LBP in Jordan. Content validation of the preliminary STarTBack–AR version showed that 9 out of the 10 items were clear and understandable to participants, with only minor modifications needed to item 5 for cultural appropriateness. Reliability and validity testing supported the use of the adapted STarTBack–AR tool for people with LBP in Jordan.

The current cross-cultural adaptation study adhered to the guidelines outlined by Beaton et al. (2000) [22]. Each step, including the composition of the translation committee, was clearly documented. However, a systematic review evaluating cross-cultural adaptations of STarT Back tool into other languages found that there was no full adherence to the translation guidelines [19].

Several cultural nuances may influence how Jordanian people interpret certain items of STarTBack, especially the psychological related items. Generally, Arab including Jordanian underreport the psychological distress due to stigmatization [40,41]. This may lead participants to avoid endorsing items related to depression and anxiety.

Another nuance relates to Language. Some phrases in the tool do not have equivalent in Arabic or they are perceived differently by Arabic speakers. For item 5 (“It’s not really safe for a person with a condition like mine to be physically active”), some participants had difficulty understanding the phrase “it is not safe” in the cultural context of Jordan, where safety often refers to security. This issue aligns with findings from other cross-cultural adaptations of the STarTBack into Arabic [20], French [42], and Danish [43]. The phrase was modified to “it is dangerous” for enhanced clarity. This highlights the importance of cultural adaptation for tools translated between languages and populations.

Internal consistency testing found acceptable Cronbach’s alpha values of 0.73 for the overall STarTBack-AR scale and 0.71 for the psychological subscale, which are consistent with values reported in the original [18] and other validation studies [19,44]. The Chinese (0.93) and Iranian (0.84) versions showed higher reliability than the current Arabic version, which may be attributed to the larger sample sizes included in those studies. Nonetheless, these acceptable values of Cronbach’s alpha indicate that the items of STarTBack-AR are related to each other [29]. Furthermore, the non-high values of Cronbach’s alpha support that there was no redundancy among items of STarTBack-AR.

Construct convergent validity was supported by moderate to high correlations between the STarTBack-AR and measures of similar constructs, including the ODI (physical disability) and PCS (pain catastrophizing). This is consistent with the Iranian and French versions, although the Iranian version demonstrated stronger correlation with ODI. Lower correlations with the HADS (anxiety/depression) and NPS (pain intensity) align with findings from Elsabbagh et al. (2018) [20]. Also, the French version showed a moderate correlation with pain intensity [44]. The low to moderate correlation between STarTBack tool and pain intensity may be attributed to differences in time frames between tools.

The STarTBack tool includes items that pertain to both physical and psychological domains [18]. AUCs supported excellent discriminative ability for physical disability and pain catastrophizing, and acceptable ability for anxiety/depression. This indicates the STarTBack-AR can differentiate patients according to functional status and mental health [45–47]. These findings are consistent with those of Jonathan C. Hill et al. (2008) and Chinese version of the tool [18,48]. In contrast, the Danish version showed lower discriminative ability for physical disability, because its assessment was conducted longitudinally rather than cross-sectionally [49,50].

There were no significant floor or ceiling effects, which enhances the tool’s responsiveness [51]. These findings align with other versions of the tool; French, Chinese, and Arabic-Saudi [20,44,48]. The adapted STarTBack-AR shows potential for accurate risk screening, treatment planning, and monitoring outcomes over time in Jordanian clinical practice. However, predictive validity was not assessed due to the cross-sectional design, which represents another limitation.

### Strengths and weaknesses

As with any study, this research has several strengths. First, it follows best practices for cross-cultural adaptation by utilizing a rigorous multi-step process as outlined by Beaton et al. (2000) [22]. This ensures semantic, idiomatic, experiential, and conceptual equivalence between the original and adapted versions. Second, it assesses multiple aspects of validity, including content, convergent, and discriminant validity, allowing for a comprehensive evaluation. Additionally, the sample size of over 100 participants is adequate for the psychometric analyses conducted [35]. The results also align well with previous validations [19], supporting the cross-cultural generalizability of findings.

However, some limitations should be acknowledged. First, while internal consistency reliability was assessed, test-retest reliability was not. Due to limited funding, we were unable to conduct a longitudinal assessment of test–retest reliability. This limits the ability to fully evaluate the tool’s reliability over time. Second, the study utilized a cross-sectional design, preventing assessment of predictive validity which is an important psychometric property to predict future outcomes. Third, while the sample size was adequate for analyses, this study used convenience sampling, recruiting participants only from hospitals and physiotherapy clinics. This may limit the generalizability of the findings to other care settings. Fourth, although online data collection allows for wider geographical coverage, it may lead to potential bias especially selection bias. People with internet access and have interest in the topic are more likely to participate. The current study presents preliminary results that require further validation in larger and more diverse samples. Also, additional psychometric testing is warranted to improve the measurement properties of the tool, including assessment of test-retest reliability and predictive validity.

### Research and clinical implications

This study has important implications for both clinical practice and research involving patients with LBP in Jordan. In clinical practice, the validated STarTBack-AR tool can now be utilized to stratify Arabic-speaking patients according to their risk of poor outcome. This will allow clinicians to better match patients with targeted treatment approaches and improving individualization of care. It may also enhance communication and shared-decision making if patients can complete assessments in their native language.

From a research perspective, further evaluation of the tool’s predictive validity and responsiveness over time is still needed. Future longitudinal studies could assess whether stratified care based on STarTBack-AR risk scores leads to better clinical outcomes for patients. Additional validation work may also help generalize findings to other Arabic dialects and Middle Eastern countries.

Overall, this cross-cultural adaptation addresses a critical gap, advancing both evidence-based practice for LBP and the development of culturally-appropriate patient-reported outcome measures across diverse populations.

## Conclusion

In summary, this study translated and validated the STarTBack for use in Jordan, addressing a significant gap in the assessment of LBP. The validated tool can be applicable in neighbor countries due to shared cultural and linguistic similarities. The adapted STarTBack-AR demonstrates reliability and validity for screening and classifying LBP patients based on risk in this population. While these finding are trustworthy, future studies assessing predictive validity are essential before recommending the tool for wider implementation.

## Supporting information

S1 File Online Package. It is the online package that was sent to the participants. It includes the consent form, demographic information, and different health outcome measures.
(PDF)

S2 File Arabic version of STarT Back Tool. It is the final Arabic version of STarT Back Tool.
(PDF)

S3 File STARTBACK Data. (XLSX)

S4 File Inclusivity in global research questionnaire. (DOCX)
