# Supplementary material for: Cross-cultural adaptation and psychometric validation of the STarT back tool for Jordanian Arabic-speaking adults with low back pain
Source: PLoS One. 2025 Nov 6;20(11):e0336398. doi: 10.1371/journal.pone.0336398 (PMC12591473; doi:10.1371/journal.pone.0336398)
Supplement: S1 File — It is the online package that was sent to the participants. It includes the consent form, demographic information, and different health outcome measures. (PDF) [file pone.0336398.s001.pdf]

S1 File: Online Package

السلام عليكم ورحمة الله،

يقوم الباحث د. أويس جعفر عليان من
جامعة عمان الأهلية بعمل بحث يهدفما إلى فحص خصائص استبيان جامعة كييل لفحص آلام الظهر باللغة العربية.
إذا وافقت على المشاركة في هذا البحث سيطلب منك تعبئة بعض الاستبانات والمعلومات الشخصية عن طريق الانترنت. لن يتعدى تعبئة النموذج اكثر من 15 دقيقة

لن يتم جمع أي معلومات تعريفية عن المشارك بالبحث كالاسم او رقم الهوية الوطنية

لمزيد من المعلومات، الرجاء التواصل مع الباحث الرئيسي وشاكرين لكم حسن تعاونكم.

د. أويس جعفر عليان

خلوي: 0778461292

بريد إلكتروني:

o.eilayyan@ammanu.edu.jo

ojeilayyan@gmail.com

الرجاء الضغط على الرابط التالي لقراءة نموذج الموافقة
https://drive.google.com/file/d/1cH0LWEExQNcTQKPCTApHZjVVducmF3eV/view?usp=drive_link

* Indicates required question

1. لقد قرأت وفهمت الموافقة المستنيرة ، وتم شرح الغرض من البحث لي ، وبالتالي أوافق على المشاركة من خلال استكمال استبيان الاستبيان وإعادته إلى الباحث *

Check all that apply.

- [ ] نعم
- [ ] لا

المعلومات الشخصية

2. تاريخ الميلاد *

____

3. الجنس *

Mark only one oval.

- ( ) ذكر
- ( ) أنثى

4. الحالة الاجتماعية *

Mark only one oval.

- ( ) أعزب
- ( ) متزوج
- ( ) مطلق
- ( ) أرمل

5. الوظيفة *

____

6. منذ متى وانت تعاني من آلام أسفل الظهر *

____

7. هل تلقيت اية علاجات لتخفيف آلام أسفل الظهر؟

Check all that apply.

- [ ] طبيب عام
- [ ] طبيب أخصائي آلام أسفل الظهر
- [ ] علاج طبيعي
- [ ] ادوية
- [ ] لا شيء

أداة جامعة كيل لفحص آلام الظهر

8. إختر الاجابة المناسبة على الأسئلة التالية بناء على آخر إسبوعين *

Mark only one oval per row.

	موافق (1)	غير موافق (0)
انتقل الم ظهري الى إحدى أو كلتا ارجلي في بعض الاوقات خلال الاسبوعين الماضيين	⬭	⬭
شعرت بألم في كتفي أو رقبتي في وقت ما خلال الاسبوعين الماضين	⬭	⬭
استطعت المشي فقط لمسافات قصيرة بسبب الم ظهري	⬭	⬭

ارتديت ملابسي أبطأ من المعتاد بسبب الم ظهري خلال الاسبوعين الماضيين	◯	◯
من الخطر لشخص يعاني مما أعاني منه ان يكون نشيط جسديا	◯	◯
تدور في ذهني افكار مقلقة في كثير من الأوقات	◯	◯
اشعر بأن الم ظهري فظيع ولن يتحسن أبدا	◯	◯
بشكل عام، لم أعد استمتع بما كنت	◯	◯

استمتع به
في السابق

9. إجمالا، ما مدى انزعاجك من ألم ظهرك خلال الاسبوعين الماضيين؟ *

Mark only one oval.

- ابدا
- قليلا
- متوسط
- كثيرا جدا
- لأقصى حد

(Oswestry Disability Index (ODI) version 2.1b) مؤشر أسويستري للعجز (ODI©Jeremy Fairbank)

هذا الإستبيان مصمّم لإعطائنا معلومات عن كيفية تأثير مشاكل ظهرك في قدرتك على إدارة حياتك اليومية.
الرجاء الإجابة على جميع الأسئلة. ضع علامة على مربع واحد فقط في كل قسم بحيث يكون الأقرب وصفًا لحالتك اليوم.

10. القسم 1 - شدة الألم *

Mark only one oval.

- ليس لديَّ أي ألم في الوقت الحالي.
- الألم خفيف جدًا في الوقت الحالي.
- الألم متوسط في الوقت الحالي.
- الألم شديد إلى حد ما في الوقت الحالي.
- الألم شديد جدًا في الوقت الحالي.
- الألم في أسوأ حالة يمكن تخيّلها في الوقت الحالي.

11. القسم 2 - العناية الشخصية (الإغتسال، إرتداء الملابس، إلخ.) *

Mark only one oval.

- يمكنني الإعتناء بنفسي بشكل طبيعي دون التسبب في مزيد من الألم.
- يمكنني الإعتناء بنفسي بشكل طبيعي ولكن هذا الأمر مؤلم جدًا.
- من المؤلم أن أعتني بنفسي وأكون بطيئًا وحذرًا.
- أحتاج إلى بعض المساعدة لكنني أستطيع التعامل مع معظم نواحي العناية الشخصية.
- أحتاج إلى المساعدة كل يوم في معظم نواحي العناية الشخصية.
- لا أستطيع ارتداء ملابسي وأغتسل بصعوبة وألزم الفراش.

12. القسم 3 - رفع الأشياء *

Mark only one oval.

- أستطيع رفع أوزان ثقيلة بدون مزيد من الألم.
- أستطيع رفع أوزان ثقيلة لكن ذلك يسبب مزيدًا من الألم.
- الألم يمنعني من رفع الأوزان الثقيلة عن الأرض ولكنني أستطيع أن أتعامل معها إذا كانت الأوزان في موضع مريح، على طاولة مثلاً.
- الألم يمنعني من رفع الأوزان الثقيلة ولكنني أستطيع أن أتعامل مع الأوزان الخفيفة إلى المتوسطة إذا كانت في موضع مريح.
- أستطيع رفع الأوزان الخفيفة جدًا فقط.
- لا أستطيع رفع أو حمل أي شيء على الإطلاق.

13. القسم 4 - المشي *

Mark only one oval.

- الألم لا يمنعني من المشي لأي مسافة.
- الألم يمنعني من المشي لأكثر من كيلومتر واحد.
- الألم يمنعني من المشي لأكثر من نصف كيلومتر.
- الألم يمنعني من المشي لأكثر من 100 متر.
- أستطيع المشي فقط بإستخدام عصا أو عكازين.
- ألزم الفراش معظم الوقت وأضطر إلى الزحف للذهاب إلى الحمام.

14. الجلوس - 5 القسم *

Mark only one oval.

- أستطيع الجلوس على أي كرسي لأطول مدة كما أشاء.
- أستطيع الجلوس على كرسيّ المفضّل لأطول مدة كما أشاء.
- الألم يمنعني من الجلوس لأكثر من ساعة واحدة.
- الألم يمنعني من الجلوس لأكثر من نصف ساعة.
- الألم يمنعني من الجلوس لأكثر من 10 دقائق.
- الألم يمنعني من الجلوس تماماً.

15. الوقوف - 6 القسم *

Mark only one oval.

- أستطيع الوقوف لأطول مدة كما أريد بدون المزيد من الألم.
- أستطيع الوقوف لأطول مدة كما أريد لكن مع مزيد من الألم.
- الألم يمنعني من الوقوف لأكثر من ساعة واحدة.
- الألم يمنعني من الوقوف لأكثر من نصف ساعة.
- الألم يمنعني من الوقوف لأكثر من 10 دقائق.
- الألم يمنعني من الوقوف تماماً.

16. القسم 7 - النوم *

Mark only one oval.

- لا يكون نومي متقطعًا أبدًا بسبب الألم.
- يكون نومي متقطعًا من حين لآخر بسبب الألم.
- أنام أقل من 6 ساعات بسبب الألم.
- أنام أقل من 4 ساعات بسبب الألم.
- أنام أقل من ساعتين بسبب الألم.
- الألم يمنعني من النوم تماماً.

17. القسم 8 - الحياة الجنسية (إن وجدت)

Mark only one oval.

- حياتي الجنسية طبيعية ولا تسبب لي مزيدًا من الألم.
- حياتي الجنسية طبيعية لكنها تسبب لي مزيدًا من الألم.
- حياتي الجنسية طبيعية تقريبًا لكنها مؤلمة جدًا.
- حياتي الجنسية مقيّدة بشدة بسبب الألم.
- حياتي الجنسية معدومة تقريبًا بسبب الألم.
- الألم يمنع أي حياة جنسية تماماً.

18. الحياة الإجتماعية - القسم 9 *

Mark only one oval.

- حياتي الإجتماعية طبيعية ولا تسبب لي مزيدًا من الألم.
- حياتي الإجتماعية طبيعية ولكنها تزيد من درجة الألم.
- ليس للألم تأثير كبير على حياتي الإجتماعية باستثناء الحدّ من أنشطتي التي تتطلب مجهودًا بدنيًا أكبر كالرياضة، إلخ.
- لقد جعل الألم حياتي الإجتماعية مقيّدة فلا أخرج غالباً.
- لقد جعل الألم حياتي الإجتماعية قاصرة على المنزل.
- ليس لي أية حياة إجتماعية بسبب الألم.

19. السفر - القسم 10 *

Mark only one oval.

- أستطيع السفر إلى أي مكان بدون ألم.
- أستطيع السفر إلى أي مكان ولكن يتسبب ذلك في مزيد من الألم.
- الألم شديد لكنني أقوم برحلات لأكثر من ساعتين.
- الألم يجعلني مقتصرًا على رحلات أقل من ساعة واحدة.
- الألم يجعلني مقتصرًا على رحلات قصيرة ضرورية تقل مدتها عن 30 دقيقة.
- الألم يمنعني من السفر إلا لتلقي العلاج.

(ODI©Jeremy Fairbank)
For any information on the use of the ODI, please contact Mapi Research Trust, Lyon, France. Internet: https://eprovide.mapi-trust.org

Pain Catastrophizing Scale (PCS) (Copyright © 1995 Michael JL Sullivan)

الكثير
من الأشخاص يعانون من آلام مختلفة في لحظات حياتهم، كآلام الصداع، آلام الأسنان، آلام المفاصل والعضلات.

لذلك، نحن مهتمون بمعرفة الأفكار
والمشاعر التي تروادك عندما تكون في حالة الألم، في مقياس الألم التالي، يوجد 13 عبارة
تصف جوانب مختلفة من الأفكار والمشاعر التي قد تتصاحب مع الألم.

يرجى
تحديد مدى انطباق هذه العبارات عليك حسب المقياس التالي

20. *

Mark only one oval per row.

	نعم =4 في كل الأوقات	نعم =3 إلى حد كبير	نعم =2 إلى حد متوسط	نعم =1 إلى حد بسيط	لا =0 على الإطلاق
أنا قلق طوال الوقت حول ما إذا كان الألم سينتهي أم لا					
أشعر أنني لا أستطيع الاستمرار فيما أشعر فيه من الألم					
الألم فظيع، وأعتقد أنه لا يمكن أن يتحسن					
الألم مروع، وأشعر أنه يثقل كاهلي و يتعبني					

أشعر أنه لا يمكنني التحمل أكثر بعد الآن					
أشعر بالخوف من أن الألم سيصبح أسوء					
أفكر دائماً بأحداث مؤلمة أخرى					
أريد زوال الألم بلهفة بالغة					
لا استطيع أن أنسى الألم					
أظل أفكر دائما في شدة الألم					
أظل أفكر باستياء عن كيفية إيقاف الألم					

ليس بوسعي فعل أي شيء للتقليل من حدة الألم	◯	◯	◯	◯	◯
أنا أتساءل عما إذا كان سيحدث شيء خطير لي	◯	◯	◯	◯	◯

PCS (Copyright © 1995 Michael JL Sullivan)
For any information on the use of the PCS, please contact Mapi Research Trust, Lyon, France. Internet: https://eprovide.mapi-trust.org

FABQ

استمارة لمعرفة مدى تأثير خوف المصابين بأوجاع الظهر السفلي من الحركة وامتناعهم عنها
فيما يلي بعض الأشياء التي أخبرنا بها المرضى الآخرون عن آلامهم. لكل عبارة ، يرجى وضع دائرة حول أي رقم من 0 إلى 6 لتوضيح مدى تأثير الأنشطة البدنية مثل الانحناء أو الرفع أو المشي أو القيادة على آلام الظهر.

21. *

Mark only one oval per row.

	غير موافق تماما (0)	1	2	غير متأكد (3)	4	5	موافق تماما (6)
**الألم سببه الحركة البدنية**	⬭	⬭	⬭	⬭	⬭	⬭	⬭
**الحركة البدنية تجعل ألمي يزداد**	⬭	⬭	⬭	⬭	⬭	⬭	⬭
**الحركة البدنية تؤلم ظهري**	⬭	⬭	⬭	⬭	⬭	⬭	⬭
**يجب أن لا أقوم بنشاط بدني كي لا يزداد الألم**	⬭	⬭	⬭	⬭	⬭	⬭	⬭
**لا استطيع عمل نشاط بدني لانه يسبب الالم و يزيده**	⬭	⬭	⬭	⬭	⬭	⬭	⬭

شدة الألم

يرجى وضع علامة على المقياس أدناه لإظهار شدة ألمك.
صفر (0) يعني لا يوجد ألم، و عشرة (10) تعني ألم شديد.

22. ما شدة ألمك الآن؟ *

Mark only one oval per row.

	0	1	2	3	4	5	6	7	8	9	10
**Row 1**	◯	◯	◯	◯	◯	◯	◯	◯	◯	◯	◯

(Hospital Anxiety and Depression Scale "HADS") مقياس القلق والاكتئاب الخاص بالمستشفى (HADS copyright © R.P. Snaith and A.S. Zigmond, copyright © Munksgaard International Publishers Ltd)

يعرف الأطباء أن المشاعر تلعب دوراً هاماً في معظم الأمراض. فإذا عرف طبيبك/طبيبتك عن هذه المشاعر،سيقدر أن يساعدك أكثر.

هذا الاستبيان مصمم لمساعدة طبيبك/طبيبتك في معرفة ما تشعر به.

الرجاء أن تقرأ كل بند فيما يلي وارسم خطاً تحت الإجابة الأقرب إلى ما كنت تشعر به في الأيام السبعة الماضية

لا تفكر طويلاً في الإجابات: إذ أن رد فعلك الفوري سيكون غالباً أدق مما لو فكرت طويلا

23. أشعر بأني متوتر *

Mark only one oval.

- معظم الوقت
- الكثير من الوقت
- أحياناً
- لا على الإطلاق

24. مازلت أستمتع بالأمور التي كنت أستمتع بها من قبل *

Mark only one oval.

- بالتأكيد
- أقل نوعاً ما مما كنت من قبل
- قليلاً
- بالتأكيد لا أستمتع

25. ينتابني نوع من الشعور بالخوف وكأن شيئاً فظيعاً على وشك الحدوث *

Mark only one oval.

- بالتأكيد وبدرجة سيئة
- نعم، ولكن ليس بدرجة سيئة
- قليلاً، ولكن لا يقلقني
- لا على الإطلاق

26. أستطيع أن أضحك وأن أرى الجانب الفكاهي في الأمور *

Mark only one oval.

- بنفس القدر كما كنت دائماً
- أقل نوعاً ما مما كنت من قبل
- بالتأكيد أقل مما كنت من قبل
- لا على الإطلاق

27. تمر بذهني أفكار مقلقة *

Mark only one oval.

- كثيراً جداً من الوقت
- الكثير من الوقت
- أحياناً
- قليلاً جداً

28. أشعر بالفرح *

Mark only one oval.

- مطلقاً
- نادراً
- أحياناً
- معظم الوقت

29. أستطيع أن أجلس مرتاحاً وأن أشعر بالاسترخاء *

Mark only one oval.

- بالتأكيد
- عادةً
- نادراً
- لا على الإطلاق

30. أشعر كأنني أحتاج إلى وقت أطول للقيام بالأشياء التي إعتدت القيام بها *

Mark only one oval.

- تقريباً كل الوقت
- معظم الوقت
- أحياناً
- لا على الإطلاق

31. ينتابني نوع من الشعور بالخوف مثل "الإضطراب" في المعدة *

Mark only one oval.

- لا على الإطلاق
- أحياناً
- كثيراً
- معظم الوقت

32. لقد فقدت الاهتمام بمنظري *

Mark only one oval.

- بالتأكيد
- لم أعد أهتم بالقدر الذي ينبغي
- ربما لا أهتم بنفس القدر
- أهتم بنفس القدر كما من قبل

33. أشعر بعدم الاستقرار وكأني يجب أن أتحرك طول الوقت *

Mark only one oval.

- كثيراً جداً
- كثيراً
- ليس كثيراً
- لا على الإطلاق

34. أتطلع إلى الأمور باستمتاع *

Mark only one oval.

- بنفس القدر مثل السابق
- أحياناً ليس بنفس القدر مثل السابق
- بالتأكيد ليس بنفس القدر مثل السابق
- بالتأكيد لا أستمتع

35. تنتابني مشاعر مفاجئة بالذعر *

Mark only one oval.

- معظم الوقت
- كثيراً
- أحياناً فقط
- لا على الإطلاق

36. أستطيع أن أستمتع بكتاب أو برنامج إذاعي أو تلفزيوني جيد *

Mark only one oval.

- كثيراً
- أحياناً
- نادراً
- مطلقاً

HADS copyright © R.P. Snaith and A.S. Zigmond, 1983, 1992, 1994.

Record form items originally published in Acta Psychiatrica Scandinavica, 67, 361–70, copyright © Munksgaard International Publishers Ltd, Copenhagen, 1983.

This edition first published in 1994 by nferNelson Publishing Company Ltd, now GL Assessment Limited,

1st Floor Vantage London, Great West Road, Brentford TW8 9AG, United Kingdom.

GL Assessment is part of GL Education www.gl-assessment.co.uk

This form may not be reproduced by any means without first obtaining permission from the publisher.

E-mail: permissions@gl-assessment.co.uk

All rights reserved including translations.

Please note that for certain countries, the HADs licensing process should go through GL Assessment. Please contact Mapi Research trust for more information.

For any information on the use of the HADS, please contact Mapi Research Trust, Lyon, France. Internet: https://eprovide.mapi-trust.org

This content is neither created nor endorsed by Google.

Google Forms
