# Supplementary material for: Cross-cultural adaptation and psychometric validation of the STarT back tool for Jordanian Arabic-speaking adults with low back pain
Source: PLoS One. 2025 Nov 6;20(11):e0336398. doi: 10.1371/journal.pone.0336398 (PMC12591473; doi:10.1371/journal.pone.0336398)

S2 File: Arabic version of STarT Back Tool

أداة جامعة كيل لفحص آلام الظهر

إسم المريض: التاريخ:

إختر الاجابة المناسبة على الأسئلة التالية بناء على آخر إسبوعين

	موافق<br>1	غير موافق<br>0
انتقل الم ظهري الى إحدى أو كلتا ارجلي في بعض الاوقات خلال الاسبوعين الماضيين		
شعرت بألم في كتفي أو رقبتي في وقت ما خلال الاسبوعين الماضين		
استطعت المشي فقط لمسافات قصيرة بسبب الم ظهري		
ارتديت ملابسي أبطأ من المعتاد بسبب الم ظهري خلال الاسبوعين الماضيين		
من الخطر لشخص يعاني مما أعاني منه ان يكون نشيط جسديا		
تدور في ذهني افكار مقلقة في كثير من الأوقات		
اشعر بأن الم ظهري فظيع ولن يتحسن أبدا		
بشكل عام، لم أعد استمتع بما كنت استمتع به في السابق		

إجمالا، ما مدى انزعاجك من ألم ظهرك خلال الاسبوعين الماضيين؟

ابدا	قليلا	متوسط	كثيرا جدا	لأقصى حد
☐	☐	☐	☐	☐
صفر	صفر	صفر	1	1

المجموع الكلي (9 نقاط): ________________ المجموع الجزئي (الأسئلة من 5-9): _______

نظام احتساب نقاط أداة جامعة كيل لفحص آلام الظهر

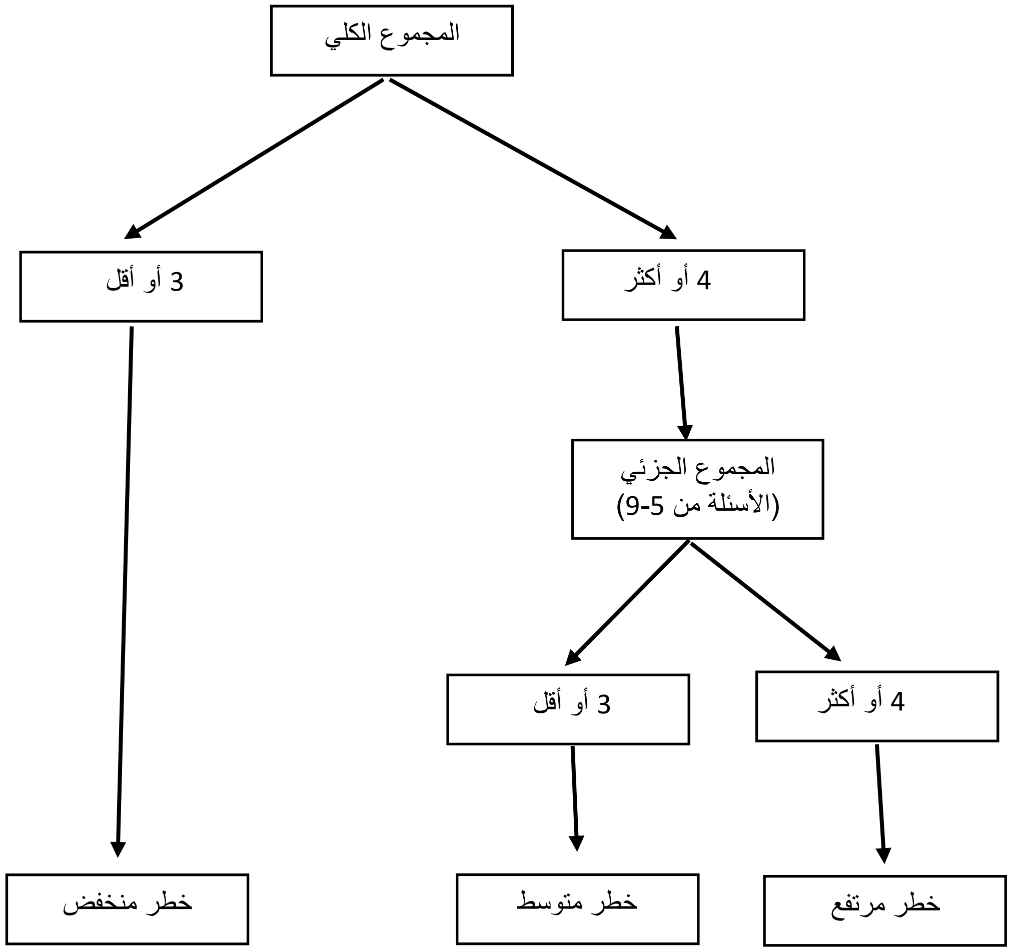

Supplement: S2 File — It is the final Arabic version of STarT Back Tool. (PDF) [file pone.0336398.s002.pdf]
